# Interventions for burnout and well-being in homelessness staff: A systematic scoping review

**DOI:** 10.1371/journal.pone.0309866

**Published:** 2025-05-21

**Authors:** Lauren Ng, Emily Adams, David Henderson, Eddie Donaghy, Stewart W. Mercer

**Affiliations:** 1 Usher Institute, College of Medicine and Veterinary Medicine, University of Edinburgh, Edinburgh, Scotland, United Kingdom; 2 Advanced Care Research Centre, Usher Institute, University of Edinburgh, Edinburgh, Scotland, United Kingdom; Public Library of Science, UNITED STATES OF AMERICA

## Abstract

**Background:**

Homelessness staff often experience high job demands, limited resources, and significant emotional strains; with high levels of burnout, stress, and trauma being common within the workforce. Despite growing recognition of these issues, limited literature exists on interventions to address them. This study aims to conduct a systematic scoping review to map and identify interventions aimed at improving well-being and reducing burnout among homelessness staff.

**Methods:**

All eligible studies needed to include an intervention addressing burnout and/or well-being in homelessness staff, published in English with primary data. Evidence sources were left open with no data restrictions. Following protocol registration, a systematic search of five electronic databases (Medline, APA PsychInfo, Global Health, ASSIA, CINAHL) and Google Scholar was conducted. Studies were double-screened for inclusion. Methodological quality was assessed using the Mixed Methods Appraisal Tool.

**Results:**

Of the 5,775 screened studies, six met the inclusion criteria: two peer-reviewed and four non-peer-reviewed publications. No studies were retrieved from Google Scholar. The included studies comprised four quantitative non-randomised designs, one randomised controlled trial, and one mixed-methods study. All included studies were complex interventions. Three were therapy-based, two included supervision, and two were one-time educational sessions. Most were conducted in the United States (n = 4), with two in the United Kingdom. The total pooled sample was 347 participants, though four studies were missing demographic data (age and gender). The studies used heterogenous measures and outcomes. Limitations included restrictions to English-only publications, potential gaps in capturing well-being measures, and a limited grey literature scope.

**Conclusion:**

There is a lack of research on well-being and burnout interventions in frontline homelessness staff. Identified studies were generally low quality, using heterogenous measures and outcomes to assess well-being and burnout, limiting the generalisability of findings. Future research should employ more robust study designs with standardised measures and outcomes.

## Introduction

Homelessness is increasing within Europe and the United Kingdom (UK), with increasing demands placed on staff working in homelessness services [1,2]. There has been growing recognition that the well-being of homelessness staff is important to provide high-quality care for PEH [3–5]. Although staff often find the work rewarding, it is acknowledged to be challenging, with the workforce facing high levels of staff turnover, stress, burnout, and secondary trauma [3,6,7].

Homelessness staff often face high job demands, limited resources, and emotional strain [7]. PEH often have complex histories–intertwined with previous or current exposure to trauma, abuse, violence, substance misuse and mental-health concerns–which can increase the risk of experiencing vicarious trauma or secondary traumatic stress for homelessness staff [8,9]. Furthermore, homelessness staff are more likely to have adverse childhood experiences compared to the general population, which may heighten their vulnerability to burnout if not supported [10]. Broader systemic issues, such as resource disparity, insufficient funding, low wages and organisational silos between professionals caring for PEH, can further hinder the ability of staff to deliver holistic care for PEH [11,12].

While research into the mental health of homelessness staff is growing, there is limited knowledge regarding the interventions that have been evaluated to address these challenges. Recent reviews have highlighted the need for further investigation into this gap [13,14].

To advance the field, an understanding of the existing research on interventions is needed. Therefore, the objective of this systematic scoping review is to map and identify interventions aimed at improving well-being and reducing burnout among homelessness staff.

## Methods

A systematic scoping review approach was adopted to answer the wider research question, namely to identify the extent and nature of existing research, and to ascertain the methodologies used to conduct these interventions.

### Study design

This scoping review was conducted in accordance to the Joanna Briggs Institute methodology for scoping reviews [15], based on Arksey and O’Malley [16] and Levac et al’s [17] framework. The review is reported using the PRISMA extension for Scoping Reviews (PRISMA-ScR) flow diagram [18]. The review protocol was registered on Open Science Framework (OSF) in May 2023 (S1 File) [19].

### Research questions

This scoping review addressed the following questions:

1. What interventions have been conducted in the homelessness sector to address staff well-being and burnout?
a. In what settings and context were these interventions carried out?
b. What measurement tools and outcomes were used to evaluate well-being and burnout in these studies?
c. How did the interventions change practice?

### Eligibility criteria

For the purposes of this review, well-being included any intervention addressing stress, burnout, job satisfaction, compassion fatigue, secondary traumatic stress, vicarious trauma, post-traumatic stress and well-being itself. These aspects have previously been identified as part of the emotional pressures faced among homelessness staff [13,14].

The inclusion criteria followed the Population, Concept, and Context criteria (Table 1). Studies were selected if they met the following three criteria: (1) the intervention specifically addressed burnout and well-being in homelessness staff and/or trainees; (2) full-text was available in the English language; and (3) the evaluation contained primary data. Evidence sources were left open, with no date restrictions.

**Table 1:** Study criteria (Population, Concept, Context and Evidence sources).

	Inclusion criteria	Exclusion criteria
**Participants**	Any support staff and trainees working in direct contact with PEH.	No direct contact with PEH
**Concept**	Any interventions that address burnout and well-being in homelessness workers.	Interventions not addressing burnout or well-being
**Context**	Any organisation supporting PEH	Organisations that do not work with PEH
**Evidence sources**	Any research assessing primary data in the English language No restrictions with source type or publication date	Non-English study, which has not been translated No primary data

### Information sources and strategy

An initial search of Medline, PsychInfo, Global Health, ASSIA and CINAHL was undertaken to identify articles relating to the review. In addition, recommended search strategies from a related systematic review and scoping review were used to supplement the initial scoping searches [13,14]. An academic librarian was then consulted to help refine the search terms and databases.

The final search strategy included five electronic databases: Medline, PsychInfo, Global Health, ASSIA, CINAHL. The search strategy was conducted on August 28^th^, 2023 by LN in English, due to language limitations of reviewers. The strategy was adapted to each database, with no date restrictions. To identify any additional studies, Google Scholar was searched using the following terms: (“burnout”) and (“homeless”) and (“staff”) and (“intervention”). The first 300 articles in the Google Scholar search to appear were included in the screening. References of the final included sources were also screened for supplementary articles, though none were identified. An example search string from Medline and PsychInfo is shown in Table 2.

**Table 2:** Example search string from PsychoInfo and Medline databases on the OVID platform.

Database	Search string
**An example search of APA PsychInfo and OVID Medline**	APA PsycInfo <1806 to May Week 3 2023>Ovid MEDLINE(R) ALL < 1946 to May 22, 2023> 1((Burnout or stress* or “emotional* exhaust*” or workload* or “vicarious trauma*” or “compassion fatigue” or “secondary trauma*” or PTSD or “post-trauma* stress” or “posttrauma* stress” or depression or “mental health” or “well-being” or wellbeing or “job satisfaction” or “job dissatisfaction” or resilience or coping or “self-efficacy”) adj4 (work* or professional* or employe* or staff or personnel* or manager*)).mp. [mp = ti, ab, hw, tc, id, ot, tm, mf, bt, nm, fx, kf, ox, px, rx, an, ui, sy, ux, mx]214144 2(homeless* or houseless* or “street dwell*” or “shelter dwell*” or “street youth*” or “street people” or “street child*” or “street person*” or unhoused or unsheltered or “rough sleep*” or “sleep* rough” or runaway* or “supported housing” or “fixed abode” or “ill-housed” or vagrant* or “people living on the street*” or “sofa surf*” or shelter*).mp. [mp = ti, ab, hw, tc, id, ot, tm, mf, bt, nm, fx, kf, ox, px, rx, an, ui, sy, ux, mx]54999 3(intervention* or program* or education or training or workshop* or course* or curriculum or approach* or service* or “random* control* trial*” or rct* or “experimental design*”).mp. [mp = ti, ab, hw, tc, id, ot, tm, mf, bt, nm, fx, kf, ox, px, rx, an, ui, sy, ux, mx]9197235 41 and 2 and 3839

### Study selection

**Fig 1:**
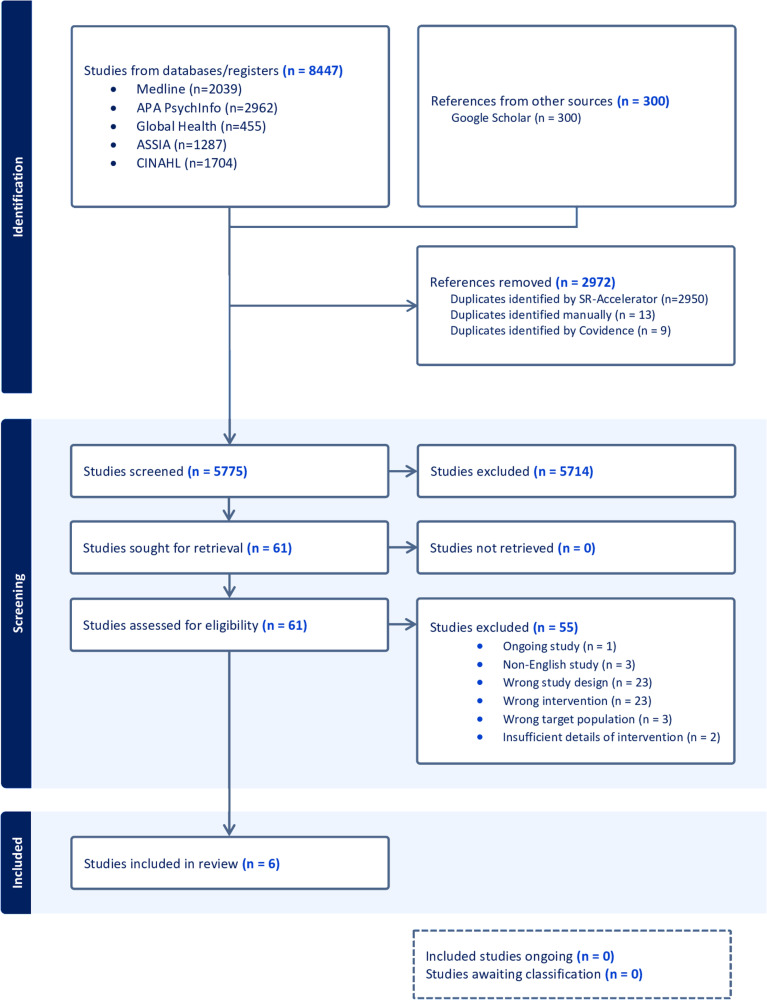
PRISMSA flowchart of scoping review.

Our search initially yielded 8,447 articles, in addition to 300 articles retrieved from Google Scholar. SR-Accelerator was used to remove any initial duplicates, with further duplicates removed by Covidence or manually by a reviewer. Search results were uploaded onto Covidence. Inclusion and exclusion criteria were completed by the primary reviewer (LN). Four reviewers (LN, EA, DA, ED) completed Title and Abstract screening independently and two reviewers (LN and EA) completed Full Text review and Data Extraction independently. Two reviewers, including the primary reviewer (LN), independently assessed the papers and identified if they met the inclusion criteria. Where there were discrepancies in study selection, a third and fourth reviewer (SM and EA) adjudicated on the final decision. Fig 1 summarises the screening process and reasons for exclusion.

### Charting the data

A structured spreadsheet was developed to systematically extract data relevant to the review’s questions and sub-questions. Two reviewers (LN and EA) independently charted data from all included studies. Extracted data was organised into three tables:

• Characteristics of included studies: (i) author, (ii) publication characteristics, (iii) setting, (iv) participant sample, and (v) methods.
• Intervention components and measures: (i) intervention name, (ii) complex intervention, (iii) components, (iv) duration, and (v) outcomes and measures.
• Key findings and future recommendations from included studies: (i) intervention name, (ii) key findings, (iii) conclusion, and (iv) future recommendations.

### Quality assessment

The quality of the final included studies (n = 6) was assessed by the primary reviewer (LN) using the Mixed Methods Appraisal Tool (MMAT) [20], chosen for its suitability in appraising a wide range of study designs. Each study was evaluated according to the MMAT criteria based on its design [20]. The assessment focused on key elements such as the clarity and appropriateness of research questions, suitability of data collection methods, completeness of outcome reporting, and consideration of potential confounding factors. Studies were rated for each criterion on a scale of “met,” “not met”, or “cannot tell” when information was insufficient to make a determination. While no studies were excluded based on quality, this assessment provided context for interpreting the strength of available evidence and highlighting limitations that may impact the reliability and generalisability of results.

## Results

After de-duplication, a total of 5,775 studies were screened. After screening, 61 studies were reviewed at full-text. Of these, 6 were eligible for the review.

### Study characteristics

The study characteristics are summarised in Table 3. Most studies were not published in peer-reviewed journals (n = 4) [21–24], with only two undergoing peer-review (n = 2) [25,26]. Among the non-peer-reviewed studies, three were dissertations published in an online database [21,23,24] and one was an unpublished manuscript from an institutional repository [22]. No included studies were retrieved from Google Scholar. Nearly all were published in the last fifteen years (n = 5) [21–23,25,26]. Most were conducted in the United States (US) (n = 4) [21,23–25], with the remaining two conducted in United Kingdom (UK) [22,26].

**Table 3:** Characteristics of included publications (n = 6).

Author	Publication characteristics	Setting	Participant sample	Methods
	1 Peer-review status 2 Type 3 Publication source (Name)	1 Service delivery setting 2 Type of service users 3 Country	1 Age (mean ± SD) 2 Gender (participant number) 3 Job role(s) 4 Total number of participants (dropouts, %)	1 Study design 2 Data collection
**Demasi (2023)**	Non-peer-reviewed Dissertation for Doctor of Nursing Practice Research Database (ProQuest)	A faith-based, non-profit organisation working with PEH PEH United States	54 years (11) Male (4) and Female (12) Volunteers 23 total (7 dropouts, 30%)	Quantitative non-randomised Cross-sectional
**Jeffrey (1999)**	Non-peer-reviewed Dissertation for Doctor of Philosophy in Psychology Research Database (Dissertation Abstracts International: Section B: The Sciences and Engineering)	Various shelter workers working with victims of domestic violence (DV) DV United States	36 years (11) 93% female A range of DV shelters workers (e.g., counsellors, program directors, administration) 267 total (195 dropouts, 73%)	Quantitative randomised control trial Cross-sectional
**Maguire et al. (2017)**	Non-peer-reviewed Unpublished manuscript Institutional Repository (University of Southampton Institutional Repository)	17 homeless organisations in London PEH (‘rough sleepers’) United Kingdom	Not stated Male (13) and Female (17) Homelessness staff 30 staff (15 dropouts, 50%)	Quantitative non-randomised Longitudinal
**Moore et al. (2019)**	Peer-reviewed Research article Academic Journal (The Clinical Teacher)	A patient-centred medical home providing healthcare, housing and social resources to veterans experiencing homelessness Veterans experiencing homelessness United States	Not stated Not stated Healthcare trainees (doctors, nurses, pharmacists and psychologists) 15 total (7 dropouts post-survey, 47% & 3 dropouts from value assessment tools, 20%, respectively)	Quantitative non-randomised Cross-sectional
**Munyoki (2022)**	Non-peer-reviewed Dissertation for Doctor of Nursing Practice Research Database (ProQuest)	A community organisation healthcare practice for low income and medically underserved populations, including PEH Vulnerable underserved populations, including PEH in a homelessness shelter site United States	Mean not stated, 60% participants were in 40–49 years age group. Not stated A range of staff (e.g., social workers and nurse practitioners) 8 total (4 dropouts, 50%)	Mixed-methods non-experimental design Not applicable
**Reeve et al. (2021)**	Peer-reviewed Research article Academic Journal (Clinical Psychology & Psychotherapy)	A homelessness organisation in East Midlands PEH United Kingdom	Not stated Female (4), no male 2 support development workers and 2 assistant managers 4 total (no dropouts, 0%)	Quantitative non-randomised Longitudinal

Information on participant demographics, namely age and/or gender, was missing in four of the six studies [22,23,25,26]. From the available data, most participants were female. Additionally, nearly all studies had small sample sizes, ranging from 4 to 30 participants (n = 5) [21–23,25,26]. Only one study [24] had a sample size exceeding 30 participants, with 267 participants, but it experienced a high dropout rate of 73%. Five studies had drop-out rates over 20%, ranging from 20% to 73% [21–25]. Although Reeve et al. study [26] had no drop outs, it had a small sample size of 4 participants. These methodological limitations limit the reliability and generalisability of findings from the included studies.

The settings where the studies took place included specialist homelessness organisations (n = 3) [21,22,26], a domestic violence (DV) shelter (n = 1) [24], a medical home for veterans experiencing homelessness (n = 1) [25], and a community healthcare organisation for underserved populations, including PEH (n = 1) [23].

Most of the included studies were non-randomised and lacked a comparator group, limiting the ability to infer causality and attribute observed outcomes to the interventions alone (n = 5) [21–23,25,26]. The most common study design was quantitative pre-experimental design (n = 4) [21,22,25,26], which refers to studies that do not use randomisation or include a control group. One study used a mixed-methods non-experimental design [23], which combines both qualitative and quantitative methods without randomisation or control groups. Only one study used a randomised control trial (RCT) design [24]. This was also the only study to include a control group [24].

### Interventions

All interventions varied in nature, with their respective components detailed in Table 4. All were complex interventions [21–26], defined as interventions consisting of several interacting components and measuring multiple outcomes [27]. Three interventions involved therapy components, namely cognitive behavioural therapy (CBT) [22], mindfulness [23], and acceptance and commitment therapy [26]. Two of the interventions comprised of educational sessions, one of which involved a session on self-care [21] and the other presenting a well-being toolkit [25]. Two of the sessions also incorporated elements of supervision in the intervention, namely feedback on secondary traumatic stress for homelessness staff [24] and psychologist supervision for CBT training [22]. Four of the six interventions completed mainly in-person [21,22,25,26]. One intervention involved delivering a mindfulness intervention through an online platform [23] and another intervention used an anonymous postal feedback survey for homelessness staff on secondary traumatic stress symptoms [24]. Most interventions were evaluated over one to three months [21,23,24,26]. The longest evaluation period was over an academic year, estimated to be approximately 8–10 months, though the exact duration was not specified in the study [25].

**Table 4:** Intervention components and measures.

Author	Intervention name	Complex Intervention (Yes/No)	Components	Duration (Months)	Outcomes and Measures Outcomes: Measures
**Demasi (2023)**	An in-person educational session on self-care activities	Yes	50-minute educational session on compassion fatigue and self-care Self-care tool box (e.g., mindfulness leaflets) – accessed as needed	1 month	Compassion fatigue, burnout and secondary traumatic stress: Professional Quality of Life V Scale (ProQOL)
**Jeffrey (1999)**	Feedback intervention on Secondary Traumatic Stress (STS)	Yes	Providing feedback regarding STS levels of staff Each individual staff member was assigned either control group (CG), Feedback Only Group (FG), or Feedback Intervention Group (FIG). CG received no feedback. FG and FIG received individual and director repots of STS. FIG additionally received a list of suggestions to address STS. Individual feedback involved statements provided to workers about their performance compared to others. Director feedback, provided only to shelter directors, included information of the shelter’s performance compared to other DV shelters and information on the measures.	2 months	Post-traumatic stress disorder (PTSD): Modified PTSD Symptom Scale^b^ Impact of PTSD: Impact of Events Scale (IES)^b^ Beliefs and schemas about self and others due to vicarious trauma: Traumatic Stress Institute Belief Scale (TSI) (Revision L) Coping skills: Coping Strategies Inventory (CSI) Implementing coping skills to help with PTSD: Assessment of Coping with Traumatic Stress (ACTS)
**Maguire et al. (2017)**	Cognitive-behavioural therapy (CBT) training and supervision package	Yes	Designed and led by clinical psychologist 4-day CBT skills workshops to increase workers skills in CBT, and reduce burnout and negative attitudes Psychologist-led supervision (in groups of three staff) in CBT training every 2 weeks for 6 months	6 months	Burnout: Maslach Burnout Inventory (MBI) Negative beliefs about clients: Staff Attitudes and Beliefs Questionnaire – 42 (SAB42)^b^
**Moore et al. (2019)**	A well-being toolkit intervention	Yes	Well-being toolkit with evidence-based tools, led by national expert (e.g., well-being practices, booklets) A ‘Wellness Room’ (quiet space) Daily gratitude practice in morning huddles Half-day workshop on toolkit use	8-10 months^a^	Burnout: MBI (only one scale used to avoid survey fatigue but scale not specified) Stress: Cohen Perceived Stress Scale Resilience: Connor-Davidson Resilience Scale Mindfulness: Five-Factor Mindfulness Scale (only two of the five subscales to avoid survey fatigue: ‘Nonreactivity to Inner Experience’ and ‘Observing’) Assessing the value of each tool: Bespoke Likert scales^b^
**Munyoki (2022)**	An abbreviated version of the 8-week Mindfulness-based stress reduction (MBSR) program	Yes	Presentation of MBSR program Mindfulness modules on online platform (at least 15–40 minutes, three times a week)	1 month	Assessing the intervention: Bespoke quantitative survey questions (Likert scales, multiple choice, ranking questions)^b^ Barriers and future recommendations for the intervention: Free-text boxes with qualitative data^b^
**Reeve et al. (2021)**	Acceptance and commitment therapy (ACT)	Yes	Project advertised in team meetings and personal communication ACT-intervention was split into three modules reflecting the three aspects of ACT triflex (‘being open’, ‘noticing’ and ‘being active’) and conducted as one-to-one workshop-style sessions	2-3 months	Burnout: Oldenburg Burnout Inventory (OLBI) Well-being: Personal well-being index (PWI) Psychological Flexibility: Comprehensive assessment of Acceptance and Commitment Therapy processes (CompACT) Idiographic personal value: An untested single-item measure asking participants to identify one meaningful value from home and one from work^b^

a The paper states the intervention took place over an academic year but no specific timeframe was indicated.

b These are non-validated measures.

### Outcomes and measures

The outcomes and measures used to assess well-being in homelessness staff are listed in Table 4.

With regards to outcomes, four studies assessed burnout [21,22,25,26]. Two studies assessed staff beliefs on self and/or others, including service users [22,24]. Two studies evaluated general well-being of staff [21,26] and two studies evaluated coping abilities [24,26]. Two interventions used bespoke questions assessing the interventions themselves [23,25]. One study used stress and resilience as an outcome measure [25] and another study used PTSD symptoms as an outcome measure [24].

Nearly all measures used to evaluate well-being were heterogenous across the six included studies, limiting their comparability. Only two studies used the same tool, the Maslach Burnout Inventory (MBI) [22,25], though Moore et al [25] used only one of the MBI scales to prevent survey fatigue. In addition, almost all studies incorporated non-validated measures in their evaluation, increasing the risk of measurement bias and potential of unreliable outcomes (n = 5) [22–26].

### Quality appraisal

A descriptive assessment was conducted using the MMAT criteria [20] to provide context on the quality of included studies (S1 Table).

Nearly all studies had significant methodological limitations, mainly due to small sample sizes, high drop-out rates, insufficient details on the study’s recruitment and methodology, use of non-validated measures, and lack of accounting for confounding variables. S2 Table provides a summary of the key limitations of each study.

Based on the MMAT criteria [20], four of the six studies scored between 0–20% in methodological quality [22–25], one study scored 40% [21], and one met all of the appraisal criteria [26]. Although one RCT evaluation was included [24], its quality was low, limiting the ability draw reliable conclusions. Specific methodological issues included improper randomisation, lack of baseline comparability between groups, incomplete outcome data, and a high dropout rate of 73% [24], which compromised adherence to the intervention. There was also insufficient information on whether outcome assessors were blinded.

No power calculations were conducted in any of the studies. While Reeve et al [26] claimed to meet the minimum requirement of three participants for establishing an effect in single-case experimental design research, they did not provide further justification or a formal power calculation to validate the adequacy of this sample size for detecting meaningful effects.

### Findings and recommendations from included studies

The findings and future recommendations from the included studies are summarised in Table 5.

**Table 5:** Summary of key findings and future recommendations from included studies.

Author	Intervention name	Key findings	Conclusion	Future Recommendations
**Demasi (2023)**	An in-person educational session on self-care activities	Statistically significant improvements in compassion satisfaction and burnout scores were seen after the intervention. No changes in secondary traumatic stress. A negative association was found between the number of self-care activities and burnout scores. Commonly used self-care tools included sleep hygiene and exercise handouts, and journals.	The study highlights the effectiveness of using an external facilitator to present evidence on individualised compassion fatigue and self-care.	Conducting a RCT Exploring if planned group self-care would have a greater impact
**Jeffrey (1999)**	Feedback intervention on STS	Providing feedback did not lead to reduced PTSD symptoms or distorted beliefs two months later. Findings are inconclusive due to lower number of participants on follow-up.	No significant effects in feedback post-treatment. General instructions for improving coping skills may not be motivating. More collaborative and individualised feedback may be more helpful.	Trialling direct, intensive interventions to prevent avoidance Setting goals to enhance social networks and communication
**Maguire et al. (2017)**	CBT training and supervision package	Burnout was significantly reduced after the intervention. No statistically significant changes in negative beliefs about PEH.	CBT training and supervision appear to be effective in reducing staff burnout. Positive change is achievable in a complex field with relatively modest financial investment.	Conducting a randomised control trial (RCT) Determining sustainability Establishing post-supervision data to distinguish if changes are attributable to supervision Linking staff and client outcomes^a^
**Moore et al. (2019)**	A well-being toolkit intervention	No statistically significant differences after the intervention. Trainees showed a preference for team well-being tools over personal ones. Tools like ‘Patient-Centered Goals’ and ‘Shared Values’ ranked highest, suggesting a common purpose and embracing essential values can reduce burnout.	Trainees emphasised the importance of team well-being tools over personal ones.	Exploring team-based approaches for well-being Determining sustainability
**Munyoki (2022)**	An abbreviated version of the 8-week MBSR program	No statistically significant tests were carried out. Participants generally responded positively to MBSR. Challenges included busy schedules, module length and forgetfulness.	There may be potential benefits of revising the MBSR to promote well-being.	Trialling group practice with a facilitator; shorter modules; and embedding modules into daily practice
**Reeve et al. (2021)**	ACT	ACT intervention can reduce exhaustion and increase work engagement. Psychological flexibility (PF) increased in all participants and reached statistical significance for two participants. Increase in alignment in work values for three participants and alignment with home values for two participants.	ACT interventions are effective for burnout.	Focusing on relationship between PF, work-engagement and behaviours among staff Reducing measures to 2–3 items to reduce participant burden

^a^Data on the impact of the intervention on clients was completed. However, the data was patchy, minimal and poor, and did not yield significant results. The methods and findings of client outcomes were not the focus of this scoping review and thus, not included in the findings.

Three studies reported statistically significant results [21,22,26]; however, methodological limitations (detailed in the quality appraisal) limit the reliability of these findings, warranting cautious interpretation. Significant reductions in burnout were observed following an in-person educational session on self-care [21], and CBT training and supervision [22]. The Acceptance and Commitment Therapy intervention significantly increased psychological flexibility in half of the participants [26]. No significant differences were seen in secondary traumatic stress levels following feedback [24] or after the well-being toolkit intervention [25]. One study did not carry out any statistically significant tests [23]. However, this was the only study that examined sustainability, rather than outcomes, and highlighted that time and workload were barriers to completing the mindfulness modules [23].

In nearly all studies, the most common recommendation was exploring the role of group interventions, rather than individual approaches (n = 4) [21,23–25]. Other recommendations included conducting randomised controlled trials to isolate the effects of the intervention (n = 2) [21,22], and determining sustainability of the intervention [22,25]. No adverse events were reported in any studies.

## Discussion

This scoping review aimed to map and identify the existing evidence on burnout and well-being interventions for homelessness staff. Of the 5,775 studies screened, six met the inclusion criteria. Only two were published in peer-reviewed research journals. The studies varied in design, with four studies employing quantitative non-randomised designs, one being an RCT, and one using a mixed-methods design. All were complex interventions, comprising multiple interacting components and targeting several outcomes. Three interventions included therapy elements, two involved single educational sessions, and two incorporated supervision components. Most were conducted in the US, with two completed in the UK. The outcome measures used across the included studies were heterogenous, limiting comparability between them. The overall quality of studies was low based on the MMAT criteria [20], with key limitations including small sample sizes, high drop-out rates, poor participant and methodological reporting, use of non-validated measures, and failure to account for potential confounders. These findings highlight the need for more robust, high-quality research to establish reliable and generalisable conclusions.

### Strengths and limitations

Strengths of this study include its comprehensive approach, involving a systematic search across five databases and Google Scholar, with no time limitations, ensuring a wide range of papers were included. The use of an academic librarian to assist in the search strategy helped reduce the bias in the search strategy. The quality assessment also provided valuable insights into the reliability of the included studies.

However, limitations include the restriction to English language publications, which may have excluded relevant studies in other languages. Due to language limitations of reviewers, it was not possible to broaden this further. Moreover, the multidimensional nature of well-being may not have been fully captured in the search strategy, potentially leaving gaps in the literature search. To mitigate this, the reviewers reviewed terms from key review papers and consulted an academic librarian for advice to ensure a wide and inclusive search scope was upheld.

The inclusion of four non-peer-reviewed studies, while providing a broader view of evidence, means findings should be interpreted with caution. The limited peer-reviewed research on this topic justified their inclusion to comprehensively map the breadth of evidence in this emerging field, in line with established scoping review methodology [16,17]. The MMAT quality appraisal was applied to contextualise these findings [20].

In addition, the inclusion of DV shelter workers, while justified by their insecure housing status [28], may have introduced some contextual differences. However, similarities between homelessness staff and DV support workers have been identified in literature, such as high work demands, burnout, compassion fatigue, and secondary traumatic stress [29]. Finally, grey literature searches were limited to Google Scholar, which may have omitted some relevant sources. While no studies from Google Scholar were ultimately included, adopting a systematic approach to grey literature searches in future reviews would help ensure all comprehensive evidence is captured [30].

### Comparison with literature

Three studies reported statistically significant findings, two of which were therapeutic interventions with relational components facilitated by clinical psychologists [22,26]. While clinical psychologists may play a role in homelessness settings, the methodological weaknesses of these studies limit the ability to draw reliable conclusions. Further high-quality research is needed to assess the effectiveness and feasibility of these approaches in homelessness settings.

The most common recommendation from studies was to explore group interventions [21,23–25]. A scoping review on vicarious trauma similarly found that group-based interventions were beneficial for fostering peer support and alleviating secondary traumatic stress [31]. The ‘Florence Nightingale effect’ suggests that staff who strongly identify with their organisation may experience lower burnout and higher job satisfaction when they view client suffering through a lens of organisational commitment, rather than a traumatic event [32]. Enhancing organisational identification through group interventions could represent a promising area for further investigation within this sector.

### Implications for research and practice

All studies employed heterogenous measures and outcomes to evaluate well-being, with only two studies using the same measurement tool and several relying on non-validated tools. The lack of standardisation prevented comparability across studies and made it difficult to assess reliability of outcomes. To improve future research, it is important to identify and agree on the most validated measure(s) for assessing well-being and burnout among homelessness staff. A Delphi exercise could facilitate consensus on the most appropriate measures for this group [33].

In addition, most studies were of low quality, with significant methodological limitations, such as poorly characterised demographics and methodologies, small sample sizes, and no power calculations. Four of the six included studies were non-peer-reviewed; therefore, findings should be interpreted with caution. The majority were also quantitatively focused with minimal qualitative insights, leaving the underlying barriers and facilitators of intervention engagement unclear [34]. Many studies also employed single-group designs with short follow-up periods, making it difficult to assess the full effects and sustainability of interventions. Existing literature has underscored that appropriate follow-up times are crucial to capture the full impact of well-being interventions [35].

Future research should prioritise strengthening the evidence base with peer-reviewed studies, robust study designs, adequate power calculations, and appropriate follow-up periods to reliably assess intervention effects. Incorporating the Medical Research Council’s complex intervention guidelines will improve study rigor and ensure interventions are developed and evaluated systematically [27]. This approach would also allow studies to better capture the complexity of intervention effects and provide more contextual evidence to improve well-being in this workforce [27].

## Conclusion

This scoping review highlights a lack of high-quality evidence on interventions to improve well-being and reduce burnout among homelessness staff. Studies were generally of low quality, with heterogenous outcome measures and significant methodological limitations, limiting the ability to draw reliable conclusions. To advance the evidence base, future research should focus on more robust study designs, including mixed methods or RCTs, with appropriate power calculations and standardised measures. Incorporating the Medical Research Council’s complex intervention guidance will enhance the development and evaluation of interventions tailored to the specific needs of this sector [27].

## Supporting information

S1 Table Results of the quality assessment using the Mixed Methods Appraisal Tool (Hong et al., 2018). S1 Table Footnotes: Abbreviations: ✓ = criteria met; X = criteria not met, CT = can’t tell due to insufficient information.
(PDF)

S2 Table Summary of *s*tudy-*s*pecific *l*imitations and MMAT *r*esults (Hong et al., 2018). (PDF)

S1 File Well-being and burnout interventions for frontline homelessness staff: A Scoping Review Protocol (registered on OSF: https://osf.io/jp5yx/). (PDF)
