# Supplementary material for: Interventions for burnout and well-being in homelessness staff: A systematic scoping review
Source: PLoS One. 2025 May 21;20(5):e0309866. doi: 10.1371/journal.pone.0309866 (PMC12094747; doi:10.1371/journal.pone.0309866)
Supplement: S1 Table — Footnotes: Abbreviations: ✓ = criteria met; X = criteria not met, CT = can’t tell due to insufficient information. (PDF) [file pone.0309866.s001.pdf]

Supplementary material

S1 Table. Results of the quality assessment using the Mixed Methods Appraisal Tool (Hong et al, 2018)

Methodological quality criteria	Study (first author, year)					
	Demasi, 2023	Jeffrey, 1999	Maguire et al, 2017	Moore et al, 2019	Munyoki, 2022	Reeve et al, 2021
**Qualitative**						
1.1 Is the qualitative approach appropriate to answer the research question?					✓	
1.2 Are the qualitative data collection methods adequate to address the research question?					×	
1.3 Are the findings adequately derived from the data?					CT	
1.4 Is the interpretation of results sufficiently substantiated by data?					×	
1.5 Is there coherence between qualitative data sources, collection, analysis and interpretation?					×	
**Quantitative randomized**						
2.1. Is randomization appropriately performed?		×				
2.2. Are the groups comparable at baseline?		×				
2.3. Are there complete outcome data?		×				
2.4. Are outcome assessors blinded to the intervention provided?		CT				
2.5 Did the participants adhere to the assigned intervention?		×				
**Quantitative non-randomized**						
3.1. Are the participants representative of the target population?	×		×	×	CT	✓
3.2. Are measurements appropriate regarding both the outcome and intervention (or exposure)?	✓		×	×	×	✓
3.3. Are there complete outcome data?	×		×	×	×	✓
3.4. Are the confounders accounted for in the design and analysis?	×		×	×	×	✓
3.5. During the study period, is the intervention administered (or exposure occurred) as intended?	✓		✓	CT	✓	✓
**Mixed Methods**						
5.1. Is there an adequate rationale for using a mixed methods design to address the research question?					×	
5.2. Are the different components of the study effectively integrated to answer the research question?					×	
5.3. Are the outputs of the integration of qualitative and quantitative components adequately interpreted?					CT	
5.4. Are divergences and inconsistencies between quantitative and qualitative results adequately addressed?					×	
5.5. Do the different components of the study adhere to the quality criteria of each tradition of the methods involved?					×	
**Overall quality score (%)**	40	0	20	0	20	100

Abbreviations: ✓ = criteria met; × = criteria not met, CT = can't tell due to insufficient information
