# Supplementary material for: Interventions for burnout and well-being in homelessness staff: A systematic scoping review
Source: PLoS One. 2025 May 21;20(5):e0309866. doi: 10.1371/journal.pone.0309866 (PMC12094747; doi:10.1371/journal.pone.0309866)
Supplement: S2 Table — (PDF) [file pone.0309866.s002.pdf]

Supporting Information

S2 Table. Summary of study-specific limitations and MMAT results (Hong et al., 2018)

Author	MMAT score	Study Limitations
Demasi (2023)	40%	● Small sample size (n=23)<br>● High dropout rates (30%)<br>● Incomplete outcome data<br>● Single setting design<br>● No control group<br>● No power calculation<br>● Failure to account for confounding variables
Jeffrey (1999)	0%	● High dropout rates (73%)<br>● Incomplete reporting of participant recruitment processes, measurement tools, and outcome variables<br>● No power calculation<br>● Use of non-validated outcome measures<br>● Non-direct intervention
Maguire et al. (2017)	20%	● Small sample size (n=30)<br>● High dropout rates (50%)<br>● Incomplete demographic information (age) and outcome data<br>● No control group<br>● No power calculation<br>● No adherence measurement; therefore, unclear if supervision consistently followed CBT principles<br>● Use of non-validated outcome measures
Moore et al. (2019)	0%	● Small sample size (n=15)<br>● High drop out rates (20%)<br>● Incomplete demographic information (age, gender) and outcome data<br>● Conducted over an academic year and unclear if changes attributable just to intervention (eg. may be related to team-building)<br>● No control group<br>● No power calculation<br>● Failure to account for confounding variables<br>● Use of non-validated outcome measures<br>● Single setting design
Munyoki (2022)	20%	● Small sample size (n=8)<br>● High drop out rates (50%)<br>● Incomplete demographic information (gender) and outcome data<br>● No power calculation<br>● Single setting design<br>● No control group<br>● Failure to account for confounding variables<br>● Use of non-validated outcome measures
Reeve et al. (2021)	100%	● Small sample size (n=4)<br>● Missing demographic information (age)<br>● Use of non-validated outcome measures<br>● No power calculation<br>● Single setting design

		• No control group
