# Supplementary material for: Interventions for burnout and well-being in homelessness staff: A systematic scoping review
Source: PLoS One. 2025 May 21;20(5):e0309866. doi: 10.1371/journal.pone.0309866 (PMC12094747; doi:10.1371/journal.pone.0309866)
Supplement: S1 File — (PDF) [file pone.0309866.s003.pdf]

Supplementary material

S1 File. Well-being and burnout interventions for frontline homelessness staff: A Scoping Review Protocol (registered on OSF: https://osf.io/jp5yx/)

Introduction

Homelessness has increased in Europe and the United Kingdom over the past decade, along with the demands on staff working in homelessness services (Tunstall, 2019; FEANTSA and Foundation Abbe Pierre, 2020).

With the rising numbers of people experiencing homelessness (PEH), there has been growing recognition that the well-being of frontline homelessness workers is essential in order to provide high-quality care for PEH (eg. Manning & Greenwood, 2018; Lemieux-Cumberlege & Taylor, 2019; 2019; Gaboardi et al., 2022). Although staff working in the homeless sector often find the work rewarding, it is nevertheless acknowledged to be challenging, with the workforce facing high levels of staff turnover and burnout (Carver et al., 2022).

Homelessness practitioners often endure high job demands, limited resources, in addition to physical and emotional health strains (Wirth et al., 2019a). PEH often have complex histories, intertwined with previous or current exposure to trauma, abuse, violence, substance misuse and mental-health concerns. Therefore, staff supporting PEH may be susceptible to vicarious traumatisation or secondary traumatic stress as a result of this exposure to trauma (Waegemakers Schiff & Lane, 2019). Moreover, broader systemic issues, such as resource disparity, insufficient funding, low wages and organisational silos among professional groups, can further hinder the ability of practitioners to provide appropriate biopsychosocial care for PEH (van den Berk-Clark, 2015; Olivet et al., 2010; Lemieux-Cumberlege et al., 2023).

While factors contributing to the mental health of frontline homelessness staff are being increasingly researched, little remains known regarding the interventions that have been carried out to address this. Pressing calls to explore this gap have been made (Peters et al., 2021; Wirth et al., 2019b). To move the field forward, an understanding of the existing interventions conducted to date is needed. Therefore, the objective of this scoping review is to map the current literature and identify existing well-being and burnout interventions that have been implemented for staff working in the homeless sector.

Review questions

This scoping review aims to provide a greater understanding on the extent, range and nature of interventions implemented to address well-being and burnout among staff working in frontline homelessness services and identify current knowledge gaps in the literature. Therefore, this review will seek to answer the following questions:

1. What interventions have been implemented in the homeless sector to address staff well-being and burnout?
    a. In what settings and context were these interventions carried out?
    b. What measurement tools were used to evaluate well-being and burnout in these studies?
    c. What were the outcomes of the interventions and how did they change practice?

Methodology

This scoping review will be conducted in accordance to the Joanna Briggs Institute (JBI) methodology for scoping reviews, based on Arksey and O'Malley (2005) and Levac et al. (2010). The six-step framework includes: (1) identifying the research question, (2) identifying relevant studies, (3) study selection, (3) charting the data, (4) collating, summarizing and reporting the results, and (5) consulting and translating knowledge (Arksey & O'Malley, 2005). The reporting of this scoping review will use the PRISMA extension for Scoping Reviews (PRISMA-ScR) flow diagram (Tricco et al., 2018).

Inclusion criteria

Table 1 specifies the Population, Concept and Context criteria of this scoping review, in addition to the included evidence sources that will be considered.

Participants	This scoping review will include any support staff and trainees working in direct contact with PEH.
Concept	The concept of this scoping review will include any interventions that address burnout and well-being in frontline homelessness workers.
Context	The context of this scoping review will be left open with no restrictions on a particular geographic location, culture, race, ethnicity, or gender. However, only studies written in the English language will be included. Furthermore, studies that do not involve staff or trainees working with PEH will be excluded.
Evidence sources	As this review aims to map the current literature, the inclusion criteria for evidence sources have been kept open to allow all types of evidence to be included.

Table 1 Inclusion criteria (Population, Concept, Context and Evidence sources)

Search Strategy

The search strategy will include all types of academic manuscripts, both published and unpublished. If a published and unpublished version of work are identified, only the published version will be included.

An initial search of Medline, PsychInfo, Global Health, ASSIA and CINAHL was undertaken to identify articles relating to the review. In addition, the recommended search strategies from a related systematic review and scoping review were used to supplement the initial scoping searches (Peters et al., 2021; Wirth et al., 2019b). Initial keywords and subject headings were identified to capture the spectrum of available literature. An academic librarian was subsequently consulted to help refine the search terms and databases.

The search strategy will be conducted in English and adapted to each database, including all keywords and subject headings. In addition, the references of all included sources will be screened for supplementary articles. If further information is required, authors of the paper will be contacted where appropriate. An example search string from Medline and PsychInfo can be found in Appendix I.

The following electronic databases will be searched to capture the relevant manuscripts across disciplines: Medline, PsychInfo, Global Health, ASSIA, CINAHL and Google Scholar. All databases will be searched with no date limitations. While no language restrictions will be imposed in the initial search, only articles published in English will be considered due to the language limitations of reviewers.

Source of Evidence Selection

After searching all respective databases, the identified manuscripts will be imported into Covidence and duplicates will be removed. The title and abstracts of the articles will be screened against the inclusion criteria by at least two independent reviewers from the authorship team. The selected articles from the preliminary screen will then undergo full text screening by at least two independent reviewers. Any disagreements relating to study eligibility will be resolved through consultation or with a third independent reviewer.

Data Extraction

Data will be extracted by two independent reviewers using a data extraction table created by the primary reviewer. A preliminary extraction table can be found in Apendix II. This tool will be modified and refined as required in the review stage. Any disagreements arising during this process will be resolved through consultation or with a third independent reviewer.

Data Analysis and Presentation

A descriptive narrative summary of the available evidence will be completed, highlighting any gaps in the literature and discussing its implications on future research and practice in the field. Furthermore, a summary table of the included studies will be included to collate the extracted evidence. Additional visual figures may be considered where appropriate.

References

Arksey, H. and O'Malley, L. (2005) 'Scoping studies: Towards a methodological framework', *International Journal of Social Research Methodology*, 8(1), pp. 19–32. doi:10.1080/1364557032000119616.

Carver, H. *et al.* (2022) 'Stress and wellbeing during the COVID-19 pandemic: A mixed-methods exploration of frontline homelessness services staff experiences in Scotland', *International Journal of Environmental Research and Public Health*, 19(6), p. 3659. doi:10.3390/ijerph19063659.

FEANTSA & Foundation Abbe Pierre. (2020). (rep.). *Fifth Overview of Housing Exclusion in Europe 2020*. FEANTSA and Foundation Abbe Pierre. Retrieved May 24, 2023, from https://www.feantsa.org/public/user/Resources/resources/Rapport_Europe_2020_GB.pdf.

Gaboardi, M. *et al.* (2022) 'Working with people experiencing homelessness in Europe', *Human Service Organizations: Management, Leadership & Governance*, 46(4), pp. 324–345. doi:10.1080/23303131.2022.2050330.

Lemieux-Cumberlege, A.H. *et al.* (2023) 'Posttraumatic stress disorder, secondary traumatic stress, and burnout in Frontline Workers in homelessness services: Risk and protective factors', *Journal of Social Distress and Homelessness*, pp. 1–12. doi:10.1080/10530789.2023.2191405.

Lemieux-Cumberlege, A. and Taylor, E.P. (2019) 'An exploratory study on the factors affecting the mental health and Well-being of Frontline Workers in homeless services', *Health and Social Care in the Community*, 27(4), pp. e367–e378. doi:10.1111/hsc.12738.

Levac, D., Colquhoun, H. and O'Brien, K.K. (2010) 'Scoping studies: Advancing the methodology', *Implementation Science*, 5(1). doi:10.1186/1748-5908-5-69.

Manning, R.M. and Greenwood, R.M. (2018) 'Microsystems of recovery in homeless services: The influence of service provider values on service users' recovery experiences', *American Journal of Community Psychology*, 61(1–2), pp. 88–103. doi:10.1002/ajcp.12215.

Olivet, J. *et al.* (2010) 'Staffing challenges and strategies for organizations serving individuals who have experienced chronic homelessness', *The Journal of Behavioral Health Services & Research*, 37(2), pp. 226–238. doi:10.1007/s11414-009-9201-3.

Peters, L., Hobson, C.W. and Samuel, V. (2021) 'A systematic review and meta-synthesis of qualitative studies that investigate the emotional experiences of staff working in homeless settings', *Health & Social Care in the Community*, 30(1), pp. 58–72. doi:10.1111/hsc.13502.

Tricco, A.C. *et al.* (2018) 'Prisma extension for scoping reviews (PRISMA-SCR): Checklist and explanation', *Annals of Internal Medicine*, 169(7), pp. 467–473. doi:10.7326/m18-0850.

Tunstall, R. (2019). (rep.). *ESPN Thematic Report on National strategies to fight homelessness and housing exclusion: United Kingdom* (pp. 6). Brussels, Belgium: European Union.

UN Habitat. (2019). (tech.). *The Strategic Plan 2020-2023*. UN Habitat. Retrieved May 24, 2023, from https://unhabitat.org/sites/default/files/documents/2019-09/strategic_plan_2020-2023.pdf.

van den Berk-Clark, C. (2015) 'The Dilemmas of Frontline Staff working with the Homeless: Housing First, discretion, and the task environment', *Housing Policy Debate*, 26(1), pp. 105–122. doi:10.1080/10511482.2014.1003142.

Waegemakers Schiff, J. and Lane, A.M. (2019) ‘PTSD symptoms, vicarious traumatization, and burnout in front line workers in the Homeless Sector’, *Community Mental Health Journal*, 55(3), pp. 454–462. doi:10.1007/s10597-018-00364-7.

Wirth, T. *et al.* (2019a) ‘“This isn’t just about things, it’s about people and their future”: A qualitative analysis of the working conditions and strains of social workers in refugee and homeless aid’, *International Journal of Environmental Research and Public Health*, 16(20), p. 3858. doi:10.3390/ijerph16203858.

Wirth, T., Mette, J., Prill, J., *et al.* (2019) ‘Working conditions, mental health and coping of staff in social work with refugees and homeless individuals: A scoping review’, *Health & Social Care in the Community*, 27(4). doi:10.1111/hsc.12730.

Appendices

Appendix I – Example of search string

The following key term string was employed in Psych Info and Medline databases in the Ovid platform.

APA PsycInfo <1806 to May Week 3 2023>

Ovid MEDLINE(R) ALL <1946 to May 22, 2023>

1 ((Burnout or stress* or "emotional* exhaust*" or workload* or "vicarious trauma*" or "compassion fatigue" or "secondary trauma*" or PTSD or "post-trauma* stress" or "posttrauma* stress" or depression or "mental health" or "well-being" or wellbeing or "job satisfaction" or "job dissatisfaction" or resilience or coping or "self-efficacy") adj4 (work* or professional* or employe* or staff or personnel* or manager*)).mp. [mp=ti, ab, hw, tc, id, ot, tm, mf, bt, nm, fx, kf, ox, px, rx, an, ui, sy, ux, mx] 214144

2 (homeless* or houseless* or "street dwell*" or "shelter dwell*" or "street youth*" or "street people" or "street child*" or "street person*" or unhoused or unsheltered or "rough sleep*" or "sleep* rough" or runaway* or "supported housing" or "fixed abode" or "ill-housed" or vagrant* or "people living on the street*" or "sofa surf*" or shelter*).mp. [mp=ti, ab, hw, tc, id, ot, tm, mf, bt, nm, fx, kf, ox, px, rx, an, ui, sy, ux, mx] 54999

3 (intervention* or program* or education or training or workshop* or course* or curriculum or approach* or service* or "random* control* trial*" or rct* or "experimental design*").mp. [mp=ti, ab, hw, tc, id, ot, tm, mf, bt, nm, fx, kf, ox, px, rx, an, ui, sy, ux, mx] 9197235

4 1 and 2 and 3 839

Appendix II

<u>Data Extraction Chart</u>

If not applicable at any point, put "NA"

Article	• Author<br>• Date of publication<br>• Article title
Type of evidence	Published or unpublished
Sample	• Age (range)<br>• Gender(s)<br>• Job role(s)<br>• Total number of participants (including dropouts)
Setting and country	• Service delivery setting (eg. outreach, community mental health team, integrated care service)<br>• Type of service users (eg. PEH, homeless veterans)<br>• Country
Intervention components	• Name of intervention (if applicable)<br>• Theoretical background<br>• Components<br>• Structure (how was the intervention carried out)<br>• Total length and duration
Outcomes and measures	• Outcomes<br>• Measurement tool(s)
Key findings	• What were the main findings?<br>• What were the implications on practice?
